# Chronic Suppurative Thyroid Abscess: A Case Report

**DOI:** 10.7759/cureus.59549

**Published:** 2024-05-02

**Authors:** Ahmed Inayath Syed, Mohan L N, Reethika Ramalingam, Puja Kumari, Niranjan Narayan

**Affiliations:** 1 Department of General Surgery, Vydehi Institute of Medical Sciences and Research Centre, Bangalore, IND

**Keywords:** e. coli, fine-needle aspiration cytology (fnac), acute suppurative thyroiditis, thyroid pathologies, open thyroidectomy, thyroid abscess

## Abstract

Thyroid abscess is a rare occurrence and is characterized by an accumulation of pus within the thyroid gland. It most commonly forms as a sequela of acute suppurative thyroiditis, and it presents as a painful swelling of the anterior neck with fever. Patients may also develop referred ear pain and compressive symptoms such as difficulty breathing and swallowing as the abscess enlarges. On examination, the swelling is often associated with erythema, local rise of temperature, and tenderness. Laboratory investigations may reveal leukocytosis, elevated acute phase reactants, and an abnormal thyroid function test. Despite advancements in diagnostic modalities and treatment approaches, literature on thyroid abscesses remains limited.

We present a unique case of a long-standing thyroid abscess resulting from chronic suppuration which did not exhibit any of the mentioned expected findings seen in other cases. This patient was euthyroid, and laboratory investigations showed no significant abnormality. It was successfully treated with total thyroidectomy and appropriate antibiotics.

## Introduction

The thyroid gland is considered to be resistant to infection due to certain features which include its rich vascular and lymphatic supply, the surrounding capsule, and a high iodine content [1]. Thus, thyroid abscess is an uncommon clinical entity with an incidence of 0.1%-0.7% of all thyroid pathologies [1]. The etiology varies from congenital anomalies to hematological spread of infection from distant sites. The most common causative agents include bacteria such as *Staphylococcus* and *Streptococcus*. Diagnostic workup involves blood investigations and radiological imaging. Thyroid abscess is treated with antibiotics and surgical intervention which may vary depending on the case.

## Case presentation

A 65-year-old female presented to the General Surgery Outpatient Department (OPD) with complaints of swelling in front of her neck for 30 years. It was insidious in onset, initially small, measuring around 2 cm x 2 cm. The patient underwent a fine-needle aspiration cytology (FNAC) test one year ago at a different hospital (the reports of which are unavailable), following which she noticed a significant increase in the size of the swelling to around 8 cm x 5 cm and discharges from the FNAC site. The discharge was spontaneous, scanty, intermittent, and seropurulent in nature. The patient also noticed a swelling over the right side of the neck for the past two years, which was nonprogressive in size. There were no constitutional symptoms or symptoms suggestive of compression, and hyper- or hypothyroidism. Her past medical and surgical histories were insignificant.

**Figure 1:**
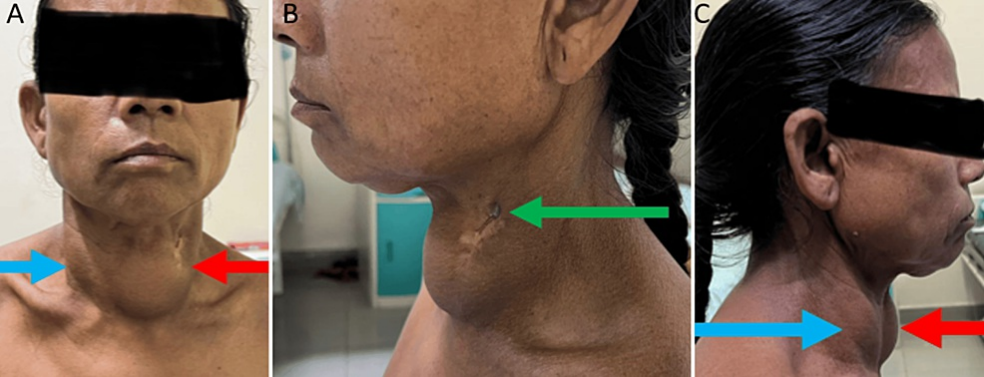
Image on the left (A: anterior view) and the right (C: lateral view) show a 65-year-old female with two discrete swellings in the region of the thyroid, the left (shown in red arrow) being larger than the right (shown in blue arrow). The image in the middle (B) shows an oblique scar on the left swelling with a continuously discharging sinus tract at the lateral end (shown in green arrow)

On examination, the patient’s vitals were unremarkable. Two discrete swellings were noted in the region of the thyroid, with the left being larger than the right (Figures 1A, 1C). The left swelling measured 8 cm x 5 cm, extending vertically from the hyoid bone to the upper border of the sternal notch and horizontally from the midline to the left sternocleidomastoid. The margins were well-defined, and the inferior border was appreciated on deglutition. It was nonfluctuant. An obliquescar of 1 cm was noted over the surface of the left swelling at the level of the hyoid (Figure 1B), showing healing by secondary intention, with a continuously discharging sinus tract at the lateral end, likely resulting from the previous FNAC. The discharge produced was scant, seropurulent, non-foul smelling, and non-blood-stained. The right swelling measured 2 cm x 3 cm. The margins were well-defined, and it extended vertically from the hyoid bone to 2 cm above the medial third of the clavicle and horizontally 2 cm from the midline to 1 cm from the right sternocleidomastoid. Both swellings were non-tender, with no local rise of temperature, and were firm in consistency, with a smooth surface. The skin over both the swellings was pinchable. Bilateral carotid pulsations were felt. There were no palpable lymph nodes. The trachea was deviated to the right. Systemic examination was within normal limits. Laboratory investigations were unremarkable as shown in Table 1.

**Table 1:** Laboratory investigations on admission BUN: Blood urea nitrogen; HIV: human immunodeficiency virus; HBsAg: hepatitis B surface antigen; HCV: hepatitis C virus

Blood investigation	Patient results	Reference values
Random blood sugar	116 mg/dL	70-140 mg/dL
Complete hemogram count (CBC)		
Hemoglobin (Hb)	11.8 g/dL	12.0-16.0 g/dL
Packed cell volume (PCV)	36.4%	36%-46%
Total white blood cell count	6,200 cells/mm^3^	4500-11,000/mm^3^
N/L/M/E/B	51.8/36.6/4.6/6.5/0.5	
Platelet count	1,39,000 cells/mm^3^	150,000-400,000/mm^3^
Erythrocyte sedimentation rate (ESR)	15 mm/hr	0-20 mm/hr
Renal function test		
BUN	10.5 mg/dL	8-23 mg/dL
Urea	22.47 mg/dL	18-46 mg/dL
Creatinine	0.59 mg/dL	0.6-1.2 mg/dL
Thyroid function test		
Thyroid-stimulating hormone (TSH)	0.76 mIU/mL	0.4-4.2 mIU/mL
Total T3	1.33 ng/mL	0.7-2.04 ng/mL
Total T4	11.93 mcg/dL	5 -12 mcg/dL
Electrolyte panel		
Sodium	139.5 mEq/L	136 -145 mEq/L
Potassium	4.79 mEq/L	3.5 -5.1 mEq/L
Calcium	9.52 mEq/L	9-11 mEq/L
Chloride	104.3 mEq/L	98-107 mEq/L
Serology (HIV, HBsAg, HCV)	Nonreactive	Nonreactive

**Figure 2:**
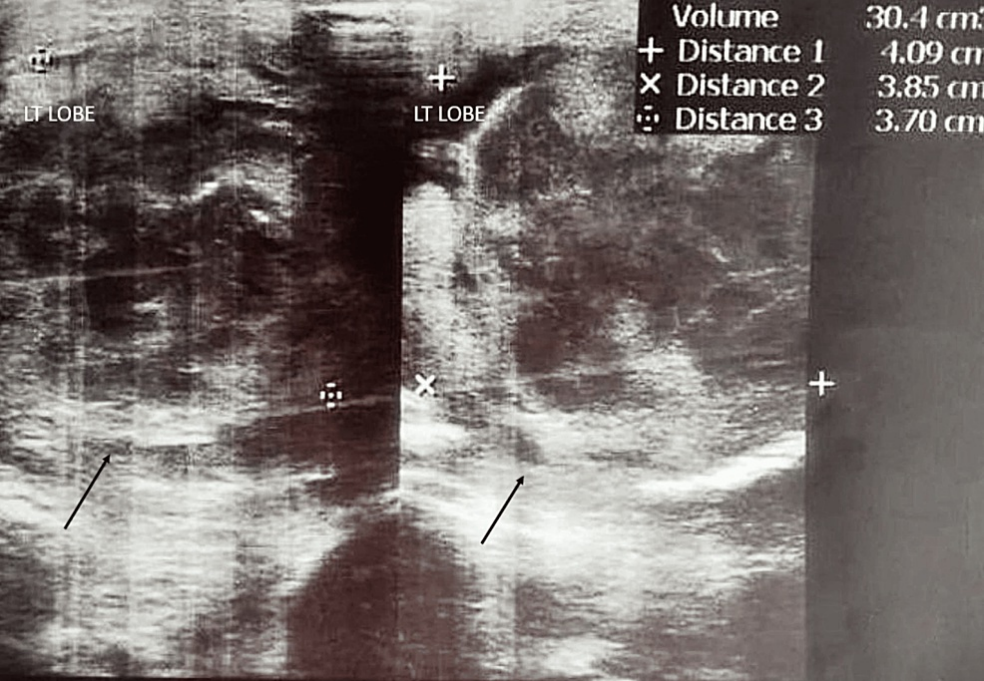
Ultrasound image showing a large heteroechoic mass in the left lobe of the thyroid containing fluid and echogenic contents, measuring 40 x 38 x 37 mm (shown in black arrow)

Ultrasound of the thyroid on admission revealed multiple nodules in the right lobe of the thyroid: two well-defined hypoechoic solid nodules with cystic components within, measuring 23 x 14 x 17 mm and 18 x 12 x 14 mm, respectively, and a few iso- to hypoechoic nodules with calcific foci, with the largest measuring 7 x 6 mm. A large heteroechoic mass was seen in the left lobe containing fluid and echogenic contents, measuring 40 x 38 x 37 mm (Figure 2) and communicating with the superficial surface via a tract, likely a thyroid abscess. Minimal vascularity was noted on Doppler interrogation. Multiple subcentimeter cervical lymph nodes were noted as well. 

**Figure 3:**
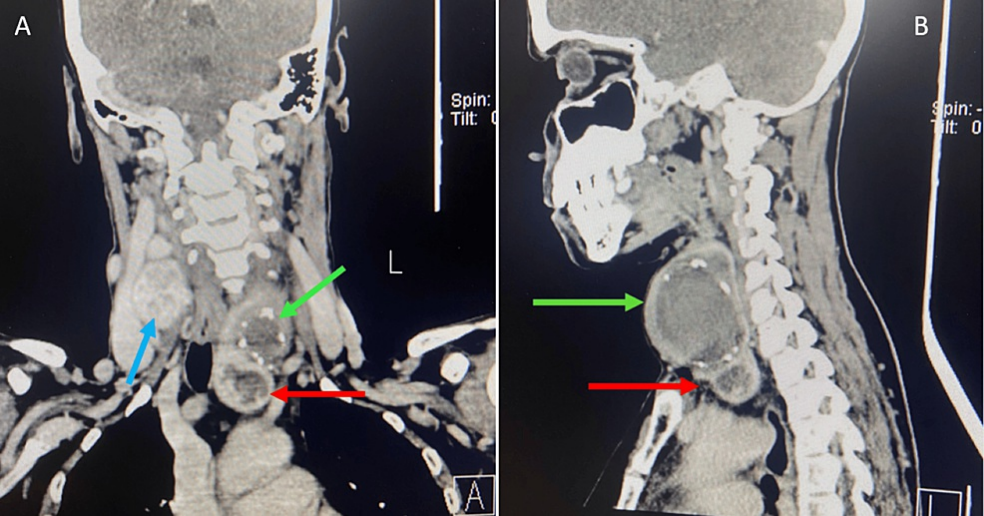
Contrast-enhanced computed tomography (CECT) scan of the head and neck showing a biloculated cystic lesion in the left lobe with calcification (shown in green arrow in A & B) and retrosternal extension into superior mediastinum (shown in red arrow in A & B). The right lobe contained a cystic lesion with multiple foci of calcifications (shown in blue arrow in A). A on the left shows the coronal view, and B on the right shows the sagittal view

**Figure 4:**
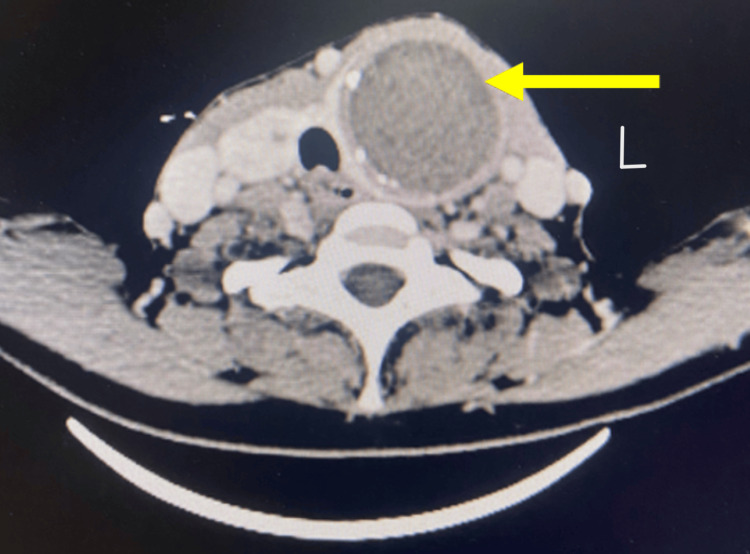
Contrast-enhanced computed tomography (CECT) scan of the head and neck showing the axial view of a cystic lesion in the left lobe with calcification (shown in yellow arrow)

**Figure 5:**
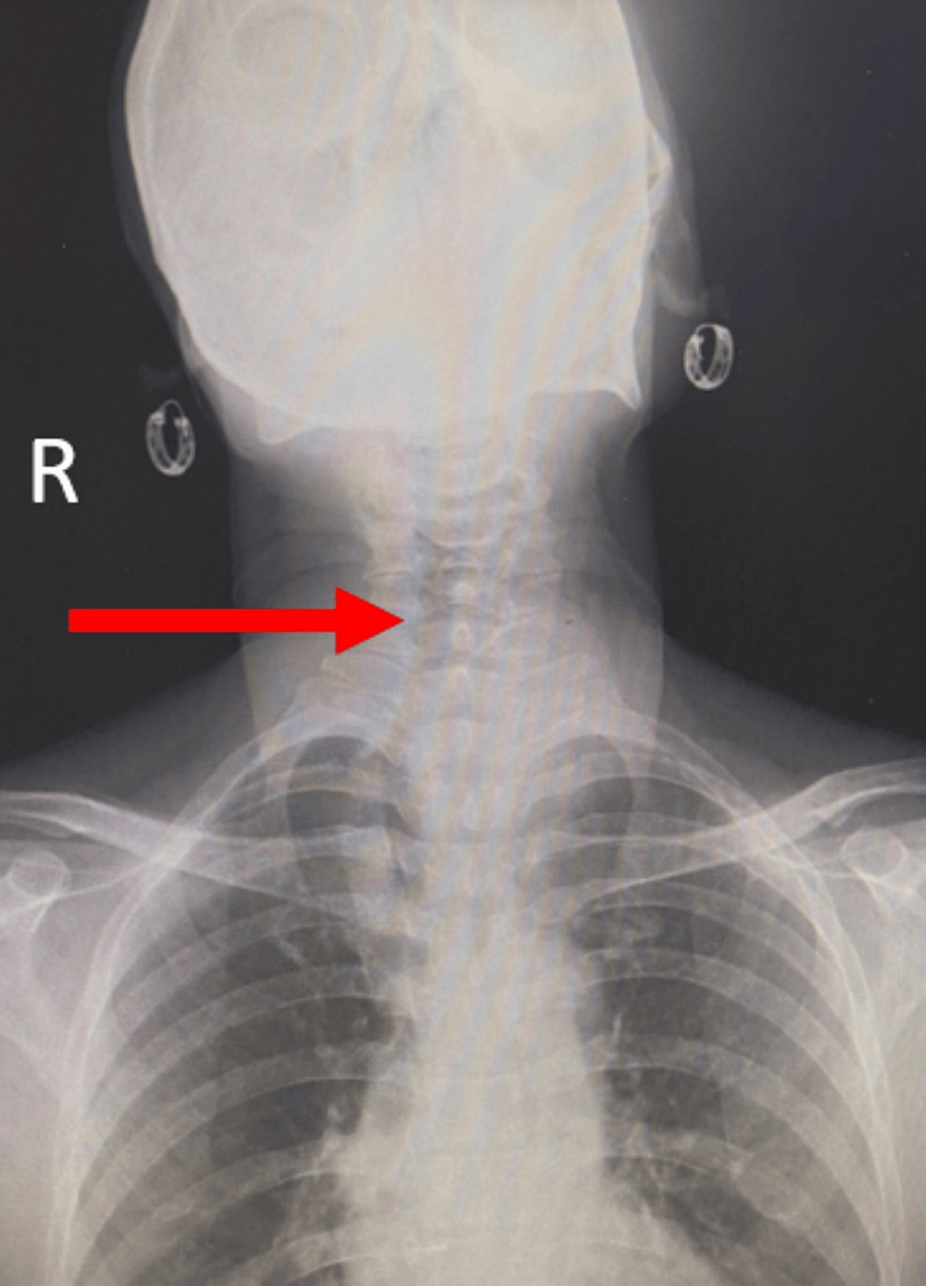
X-ray showing tracheal deviation to the right (as shown in red arrow)

Subsequently, a contrast-enhanced computed tomography (CECT) scan of the head and neck and an X-ray were done. CECT of the head and neck (Figures 3A, 3B, 4) showed the left lobe of the thyroid containing a biloculated cystic lesion with hyperdense contents within and wall calcifications, the largest measuring 42 x 44 x 48 mm: 49 cc. The lesion extended into the superior mediastinum up to the superior border of the D2 vertebra. It is seen abutting the left carotid sheath and displacing it laterally. Medially, it is seen abutting the trachea and esophagus, displacing it to the right. The right lobe showed a solid cystic lesion with hyperdense contents within and enhancing solid component, with a thick enhancing wall measuring 23 x 25 x 15 mm with multiple foci of calcifications. On-table sinogram showed a fairly well-defined, hypodense tract seen extending from the cutaneous surface of the left lateral aspect of the neck (at the level of the C5-6 vertebrae), traversing through the intermuscular plane (maximum length measuring ~36 mm and maximum width measuring ~6.6 mm) and was seen communicating with the superior cystic lesion in the left lobe of the thyroid gland, suggestive of a fistulous tract. X-ray showed deviation of the trachea to the right (Figure 5).

Ultrasound-guided FNAC of the left swelling revealed granular necrotic debris with neutrophils, few lymphocytes, and macrophages, suggestive of an abscess. FNAC of the right swelling revealed features suggestive of benign follicular nodule favoring colloid goiter. The right lobe of the thyroid was graded as Bethesda category 2. Aspirated fluid from the FNAC of the left swelling was sent for culture and the organism isolated was *Escherichia coli* (*E. coli*). No pus cells were seen.

**Figure 6:**
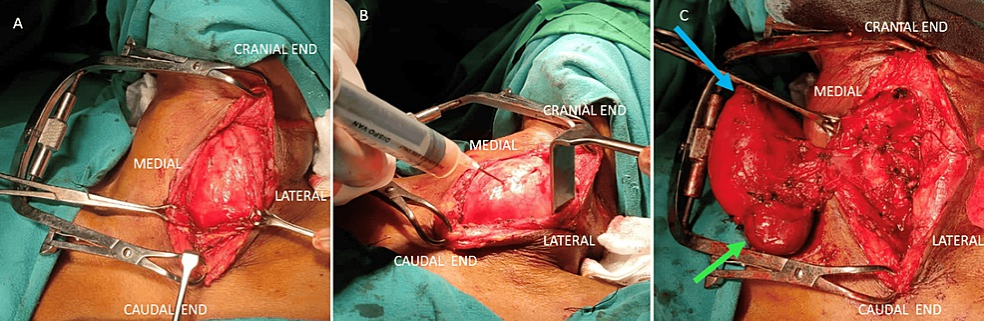
A (left image) shows the Kocher's incision placed and dissection carried out to expose the thyroid gland. B (middle image) shows on-table aspiration of purulent fluid. C (right image) shows mobilization of the left lobe (shown in blue arrow) along with its retrosternal extension (shown in green arrow)

**Figure 7:**
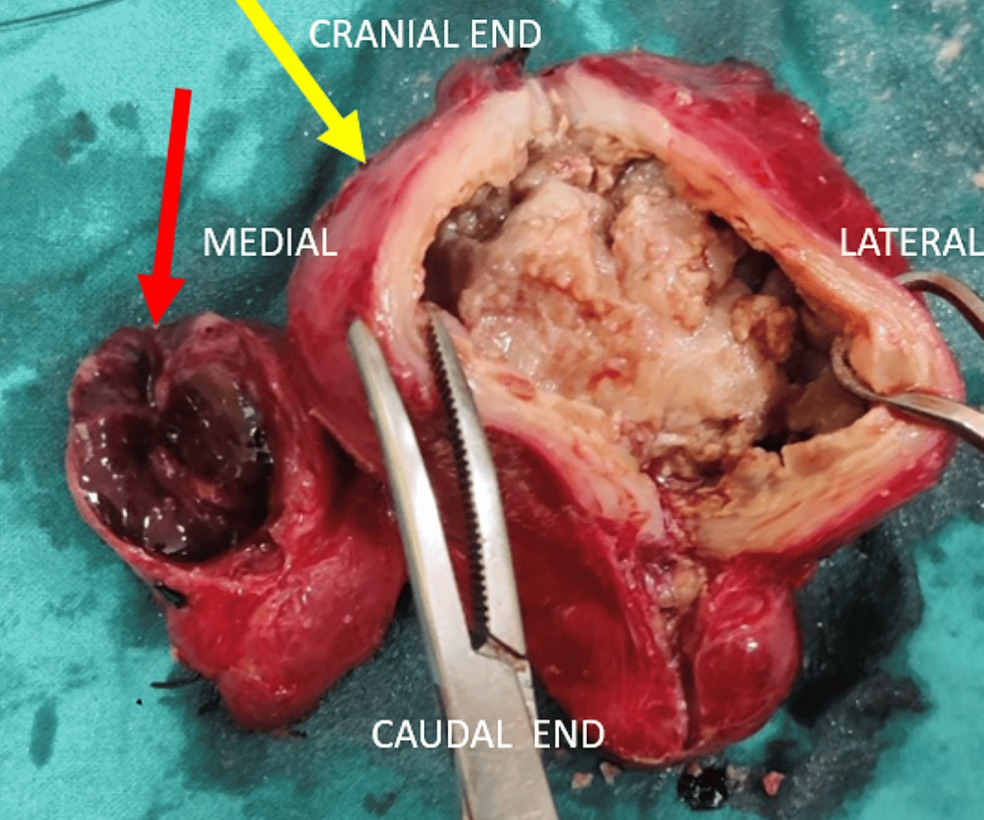
Right lobe showing congestion and multinodularity (smaller mass shown in red arrow). Left lobe showing a thick-walled cavity with caseous and purulent material (larger mass shown in yellow arrow)

The patient underwent sinus tract exploration along with total thyroidectomy. Under general anesthesia, methylene blue was injected into the sinus, and it was seen to be lost to the larger swelling on the left. The sinus was probed to find that it extended into the left lobe of the thyroid. A Kocher’s incision was placed. Subplatysmal flaps were raised, deep fascia was opened, and strap muscles were retracted to expose the thyroid gland as shown in Figure 6A. On-table aspiration was carried out (Figure 6B), and 10-15 ml of orange-brown purulent fluid was aspirated. The left lobe was mobilized first along with its retrosternal extension (Figure 6C), followed by the isthmus and the right lobe. The fistula was excised. All three parts of the thyroid were sent for histopathological examination. Hemostasis was achieved and the field was irrigated. A suction drain was placed and anchored to the skin. The incision was closed in layers. Dissection of the excised lobes carried out showed a multinodular and congested right lobe and a thick-walled left lobe containing caseous and purulent material (Figure 7).

The patient was given intravenous ceftriaxone postoperatively. The drain site and incision were healthy with no signs of infection. Seropurulent fluid collected in the drain which subsequently reduced. Daily dressing was done with removal of the drain on postoperative day 2. Levothyroxine 75 mcg was prescribed, and the patient was discharged on postoperative day 5 with no additional symptoms.

**Figure 8:**
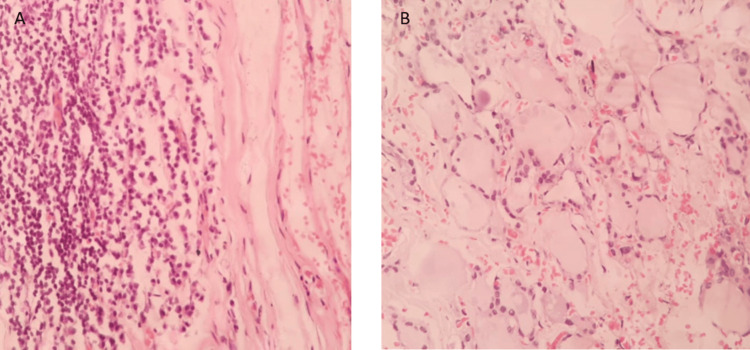
Histopathological image of the left lobe of the thyroid showing aggregates of lymphocytes amidst fibrosis, lined by necrotic debris(A, image on the left). Peripheral rim of the thyroid tissue is seen (B, image on the right)

**Figure 9:**
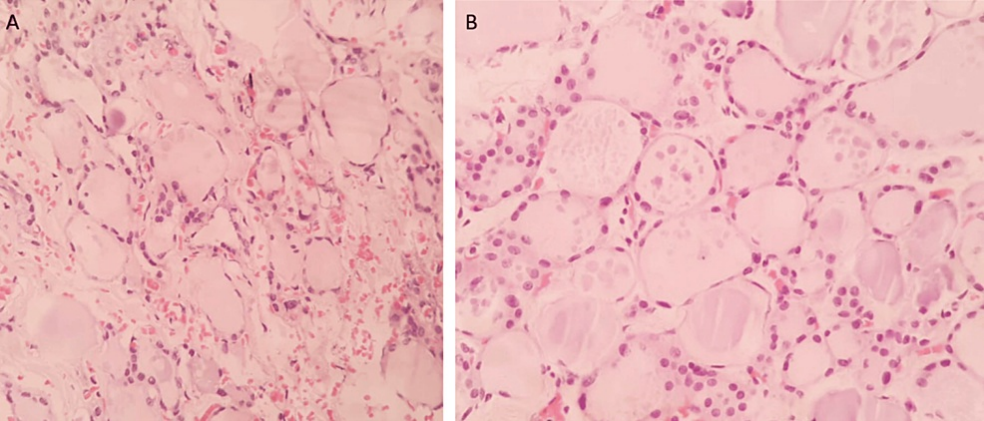
A (image on the left) is a histopathological image of the isthmus showing variable-sized dilated follicles lined by flattened follicular cells and filled with colloid. B (image on the right) is a histopathological image of the right lobe showing normal thyroid follicles with variable-sized dilated follicles lined by flattened follicular cells filled with colloid

Histopathological examination of the left lobe (Figure 8A, 8B) showed a peripheral rim of thyroid tissue, lined by necrotic debris. The cyst wall was thick and fibrosed with aggregates of lymphocytes amidst the fibrosis. The isthmus (Figure 9A) was capsulated and contained variable-sized dilated follicles lined by flattened follicular cells and filled with colloid. The right lobe (Figure 9B) was a capsulated tissue bit showing normal thyroid follicles. Also noted were variable-sized dilated follicles lined by flattened follicular cells filled with colloid.

The patient’s follow-up investigations revealed that she was persistently hypothyroid for three months postoperatively, and hence, the dosage of levothyroxine was increased from 75 mcg to 100 mcg. She also had a period of transient hypocalcemia for four months and was prescribed calcium supplements 500 mg once a day. Her parathyroid gland spontaneously resumed normal functioning, which was reflected in her calcium levels returning to normal values. There was no recurrence of symptoms. Postoperative laboratory investigations from three months after the procedure are shown in Table 2.

**Table 2:** Postoperative laboratory investigations from three months after the procedure

Blood investigation	Patient results	Reference values
Thyroid function test		
Thyroid-stimulating hormone (TSH)	9.12 mIU/mL	0.4-4.2 mIU/mL
Total T3	1.37 ng/mL	0.7-2.04 ng/mL
Total T4	6.22 mcg/dL	5-12 mcg/dL
Electrolyte panel		
Sodium	137 mEq/L	136-145 mEq/L
Potassium	4 mEq/L	3.5-5.1 mEq/L
Calcium	6.9 mEq/L	9-11 mEq/L
Chloride	103 mEq/L	98-107 mEq/L

## Discussion

Thyroid abscess is a rare occurrence with an incidence of 0.1%-0.7% of all thyroid pathologies, owing to certain features of the thyroid gland that protect it from infection. These features include its rich vascular and lymphatic supply, the surrounding firm capsule, and a high iodine content within the gland [1]. 

It most commonly occurs as a sequela of acute suppurative thyroiditis (AST) which is frequently seen in children with anatomical abnormalities such as a pyriform sinus fistula [2] and thyroglossal duct cyst [3] (tend to present earlier in life) and in adults with pre-existing conditions such as Hashimoto’s thyroiditis and thyroid malignancy [1]. Immunocompromised patients and those with a history of trauma such as FNAC and foreign body trauma from esophageal perforation are also at an increased risk of developing AST and thyroid abscess [4-6]. Infection may occur via lymphatic or hematogenous spread from distant sites, and organisms isolated include *Staphylococcus*, *Streptococcus *(accounting for 70 % of cases) [4], *Klebsiella*, *Salmonella*, *Mycobacterium*, and fungal infections in immunocompromised patients [7]. *E. coli*, the isolated organism in this case, is a rare pathogen of thyroid abscess, and only a handful of cases have been reported, most of them occurring due to hematogenous spread of partially treated urinary tract infections [7].

Patients usually present with painful neck swelling with fever, cervical lymphadenopathy, and compressive symptoms such as difficult and painful swallowing, difficulty breathing, ear pain, and hoarseness of voice. This patient’s presentation of thyroid abscess is unique because the abscess did not seem to progress or develop from AST and there were no symptoms of a typical abscess either. The patient did not report any symptoms of fever, pain, redness, referred pain, or compressive symptoms. This is also evident from the prolonged period between the onset of discharge from the swelling and her presentation to the OPD.

The only identifiable risk factor for developing thyroid abscess in this case was the FNAC done one year ago. This patient did not have any congenital anomaly or immunodeficiency conditions. A case report and review of literature published in 2020 by Htet et al. shows that around 10 cases of AST have been reported as a complication of FNA [5]. The existing research indicates that the development of acute bacterial suppurative thyroiditis from FNA can occur within a span of several days to three months. Cases involving immunocompromised patients or infections caused by less virulent organisms often exhibit a prolonged, less noticeable onset or mild symptoms [5]. The index case presented to the clinic one year after her FNAC procedure with the sole complaint of discharge from the swelling, thus implying that she did not undergo a period of AST, and the abscess is a result of chronic suppuration.

Investigations frequently reveal leukocytosis and elevated acute phase reactants. Patients may be euthyroid or may present with features of thyrotoxicosis if the abscess has occurred secondary to AST [1]. The blood investigations in the index case showed no such significant abnormality; our patient was euthyroid. Radiological investigations include ultrasound, X-ray, and CT scan to assess the extent of the lesion prior to surgery [7]. Furthermore, pus aspirated from the abscess should be sent for culture and sensitivity testing to administer effective antibiotics. Pus cultures from our patient were positive for *E. coli,* and antibiotics were administered according to the sensitivity report. *E. coli* is a rare pathogen of thyroid abscess, and only a handful of cases have been reported [7].

FNAC and biopsy are necessary to confirm the diagnosis and rule out malignancy. The histopathological examination of the left lobe tissue in this case was suggestive of a thyroid abscess resulting from chronic suppuration, aggregates of lymphocytes amidst the fibrosis, as opposed to acute suppurative thyroiditis which is more frequently the cause of a thyroid abscess. Most case reports have revolved around acute suppuration and subacute thyroiditis with a sequela of thyroid abscess. An extensive literature search on chronic suppuration or long-standing thyroid abscesses did not yield any results.

Treatment requires intravenous antibiotics and surgical intervention which may range from incision and drainage to thyroidectomy. Minimally invasive procedures such as ultrasound-guided aspiration may also prove sufficient [8]. Patients require follow-up visits for thyroid function tests and should be observed for persistence or recurrence of symptoms and postoperative complications such as parathyroid gland destruction, vocal cord palsy, etc.

## Conclusions

Thyroid abscess is a rare clinical entity, mostly developing from acute suppurative thyroiditis. Literature regarding long-standing abscesses resulting from chronic suppuration, such as this case, remains elusive. Furthermore, thyroid abscess occurring as a complication of FNA with *E. coli *as the causative agent is uncommon. One should include chronic suppurative thyroid abscess in the differential diagnosis for patients with a similar presentation.
